# Effect of continuous high thermal processing on physico-chemical and nutritional alterations in cold-pressed and refined edible oils

**DOI:** 10.3389/fnut.2026.1949768

**Published:** 2026-10-23

**Authors:** Venkatesh Nandipeta, Sai Lakshmi Chava, Nagaraju Mergu, Ananthan Rajendran

**Affiliations:** 1 Food Chemistry Division, ICMR-National Institute of Nutrition, Hyderabad, India; 2 Clinical Epidemiology, ICMR-National Institute of Nutrition, Hyderabad, India

**Keywords:** cold-pressed oil, fatty acid profile, high thermal processing, oil quality, oxidative stability, refined oil

## Abstract

**Background:**

Continuous high thermal processing of edible oils induces significant physicochemical and nutritional alterations, influence their safety, stability, and consumer acceptability. Cold-pressed oils are generally perceived as nutritionally superior, but their oxidative stability under continuous heating remains less understand compared to the refined oils.

**Methods:**

Twelve edible oils (7 cold-pressed and 5 refined oils) were collected and subjected to the continuous high thermal processing at 230 ± 5 °C for 0, 1, 2, and 4 h. Fatty acid composition, peroxide value, saponification value, free fatty acid percentage, acid value, iodine value, conjugated dienes, conjugated trienes, refractive index and colour were analysed.

**Results:**

Among the oils available in the both cold-pressed and refined types, cold-pressed groundnut, sunflower, rice bran and palm oil retained more polyunsaturated fatty acids at 1 h, but showed greater losses after 4 h then their refined types. Soybean oil exhibited a sharp rise in trans fatty acids (0.76 to 5.88%), whereas, coconut oil remained lowest (0.38%) at 4 h. Peroxide value increased in refined sunflower oil (7.60 to 15.81 mEq O_2_/kg), but refined and cold-pressed rice bran oil declined to 0.01 mEq O_2_/kg in after 2 h. Free fatty acid and acid values decreased variably across oils. Iodine value reduction was highest in coconut oil and minimal in soybean oil (6.72%). Saponification peaked in coconut oil (321 ± 0.68 mg KOH/g) and lowest in cold-pressed sunflower oil (213 ± 0.83 mg KOH/g). Conjugated triene rose steadily but remained below diene, while progressive darkening (L* reduction) was observed in all oils. Heating for 1 to 2 h impacted the saturation of fatty acids, while 4 h can increase the risk of *trans* fatty acid and dienes/trienes formation.

**Conclusion:**

Continuous high thermal processing compromises the nutritional and oxidative stability of edible oils. Coconut oil showed minimal alterations, while soybean oil and sesame oil were highly susceptible. These findings highlight the need for cautious thermal application in cooking practices to preserve oil quality.

## 1 Introduction

Oils are mixture of organic molecules (glycerol and fatty acids), derived from the seeds (nuts, grains) and fruit of plants (1). Edible oils serve as good source of hydrophilic antioxidants (e.g., tocopherol, phenolic compounds, phytosterol), which support the metabolism of fat-soluble vitamins (2). The two essential fatty acids (EFAs), omega-3 and omega-6, which cannot be synthesised by the human body, are supplied by edible oils in the diet. In addition to the EFAs, edible oils provide twice the energy than carbohydrates and protein for the same quantity, and they also infuse flavour and colour to the food (3). The oils, including groundnut (*Arachis hypogaea*), mustard (*Brassica juncea*), sesame seed (*Sesamum indicum*), sunflower (*Helianthus annuus*), soybean (*Glycine max*), coconut (*Cocos nucifera*), palm (*Elaeis guineensis*) and rice bran (*Oryza sativa*) are notable (4). Oils produced using mechanical or wood press at low temperatures, typically below 50 °C, are referred to as cold-pressed (CP) oils. These are purified by settling, filtering, and centrifugation without the use of chemicals. This method preserves nutrients, tocopherols, sterols, and phenolic compounds. The presence of natural antioxidants and phenolic compounds in oils exert greater resistance to oxidation upon heating and spoilage of the oil. Refined (RF) oils are extracted using high temperatures (≥200 °C) and then undergo chemical processing steps, including degumming, neutralization, bleaching, and deodorization, to improve shelf life. In terms of preserving the nutritional properties, cold pressing ranks higher than the refining process (1). Before being marketed, the majority of the vegetable oils were exposed to the refining process, and this commercial process converts the original oils in to products with desirable characteristics while reducing the tendency towards rancidity (5).

Vegetable oils are extensively utilised in culinary practices across the globe. One of the most common techniques is deep frying, where food is completely submerged in hot oil maintained at temperature at ≥200 °C (6). This process can lead to both beneficial and undesirable effects, depending on the specific temperature applied and the length of thermal exposure (7). Thermal processing of oils leads to hydrolysis, oxidation, polymerisation, cyclisation and isomerization, which adversely affect their physicochemical, nutritional and sensory properties (8). During processing period, oxidation of edible oils results in rancidity, generation of toxic substances, peroxides, hydroperoxides and off-flavour (9). The high levels of free fatty acids, especially linoleic acid, are undesirable in finished oils because they can cause off-flavours and shorten the shelf-life of oils. Lipid oxidation in edible oils not only alters sensory attributes such as taste, odour, and colour but also leads to the accumulation of toxic substances that can pose significant health concerns (10). Oils with higher levels of unsaturated fats are more prone to autooxidation, particularly above 180 °C, resulting in nutritional degradation. Heating of oils accelerate oxidation, reduce natural antioxidants, and increase harmful reactive oxygen species (11). Oxidative stress in vegetable oils often involves in free radical generation, which leads to the formation of primary and secondary oxidation products. Secondary oxidation products can form during the heating process, include volatile compounds such as alcohols, aldehydes, ketones, acids, etc., as well as non-volatile compounds, such as carbonyl or fatty acid dimers, trimers, and cyclic polymers, may pose a health risk to humans. Volatile compounds may be lost during the frying process; however, non-volatile polar compounds in the cooking oil are potentially hazardous as they remain in the oil, which are absorbed by food then ingested (12). Elevated levels of the polar compounds in thermally degraded oils have been associated with endothelial dysfunction and hypertension (13). Moreover, such oxidative products can also damage cell membrane and DNA in consumer, which may be involved in the ageing process and carcinogenesis (14).

Previously, Omara et al. (15) has studied changes in physicochemical properties of different brands of selected edible oils in deep-frying at 140 °C. Results have demonstrated that hard oils (coconut oil, palm oil, etc.) withstand significantly longer duration than soft oils (soybean, sunflower, etc.). Mukhametov et al. (16) investigated the effect of thermal treatment at 40 and 70 °C for 80 min, for oxidative stability of edible oils, which result in the accelerate oxidative degradation and compromise their nutritional quality. Ansorena et al. (17) conducted an experiment on the oil quality (at 65 °C and 180 °C for 90 min), reported the greatest alteration in the peroxide value and quality parameters of oils. The impact of high temperature on the fatty acid profile was studied by Cherif et al. (18), which resulted in the reduction of unsaturated fatty acid and increased in the saturated fatty acid. Nuriddinov et al. (19) assessed the impact of heat treatment on the molecular composition of the sunflower oil at >180 °C for 2 h, resulted increase in the trans fatty acid and saturated fatty acid and decline in the unsaturated fatty acid. According to Venkatesh et al. (20), prolonged exposure of oils to high temperature can increase the content of saturated and trans fatty acids and reduce that of unsaturated fatty acids. Bhardwaj et al. (21) was reported the rise of trans and saturated fatty acids and reduction of unsaturated fatty acid content in oils (refined soybean oil, groundnut oil, olive oil and rapeseed oil) at frying temperature. Degradation of EFAs, and the formation of trans fatty acids on thermal exposure of oils have been studied by Nagy et al. (22). Zahir et al. (23) investigated the physicochemical properties of mustard oil after frying at 140 and 170 °C. Results suggested that, heating gradually lessens the quality of oils and found the presence of secondary oxidative compounds in mustard oil. In quality control, the properties include refractive index, colour, free fatty acid, saponification value, peroxide value, acid value, iodine value, conjugated dienes and conjugated trienes, which are used to determine the shelf-life quality (24). Obesity, diabetes and coronary heart disease is rapidly rising in the world, especially in developing countries (25). Dietary habits, particularly the use of edible oils, play a crucial role in the pathogenesis, management, and prevention of heart diseases (26). Prolonged thermal exposure causes chemical changes, produces free radicals and diminishes nutritional value, while also forms toxic compounds in oils, which harm to health (11, 27).

Hence, judicious selection of an appropriate oil is critically important to maintain good health, as exposure of edible oils to elevated thermal conditions induce substantial alterations in oil quality parameters. However, the majority of food products (breakfast and snack items) are subjected to frying process at elevated thermal conditions, often exceeding the present reported frying temperatures (180–220 °C) (28). Despite widespread use of such a high temperature, limited research available in physicochemical and nutritional properties of various cold pressed and refined oils, particularly regarding the suitability for continuous high temperature cooking, and cold pressed oils are often marketed and perceived by consumers as nutritionally superior than refined oils, yet their behavior under prolonged thermal stress has not been reported. Therefore, this study investigated thermal degradation kinetics of twelve edible oils, including seven cold pressed oils (groundnut, sunflower, rice bran, palm, mustard, sesame, and coconut oil) and five refined oils (groundnut, sunflower, rice bran, palm and soybean oil) under controlled continuous heating. Diverse range of oils was selected to represent most widely consumed edible oils in Indian diet, spanning a broad range of saturation profiles from highly saturated (coconut and palm oil) to highly polyunsaturated (soybean, sunflower and sesame oil) oils. The novelty of this study is that it compares broad range of widely utilized edible oils, seven cold-pressed and five refined, under an identical, time resolved, continuous high thermal processing. It is essential to develop a comprehensive understanding of how oils respond to high thermal exposure across varying duration, thereby generating new insights into their thermal stability, degradation mechanism and nutritional consequences of CP and RF oils.

## 2 Materials and methods

### 2.1 Sample collection

A total of twelve commonly consumed cooking oils, those seven CP oils, including groundnut, sunflower, rice bran, palm, mustard, sesame and coconut oil, and five RF oils, namely groundnut, sunflower, rice bran, palm and soybean oil were purchased in January 2025, from three different local markets of Hyderabad, Telangana, India. The oils in these markets were sourced from different regions and suppliers, ensuring variability in brands. The selected markets were also frequently visited by the local population to purchase oil. For each oil type, three different commercial brands were purchased from each local market, this sampling yielded nine individual commercial units per oil type. These nine units were pooled into a single composite sample, which served as the sole source material for subsequent thermal treatments and analysis. The findings of this study therefore reflect the thermal response of the composite sample constructed for this experiment, not the variability of individual commercial products or brands.

### 2.2 Chemicals and reagents

The chemicals, potassium dichromate and ethanol (99.99%), were obtained from Changshu Hongsheng Fine Chemical Co., Ltd. Potassium hydroxide (KOH) (85%), potassium iodide (KI) (99.5%), hydrochloric acid (HCl) (37%), chloroform (99.8%), diethyl ether (99.5%), iodine monochloride (>97%), phenolphthalein indicator (pH 8.2–9.8) and iso-octane (99.5%) were purchased from Sigma Chemical Co., St. Louis, MO, USA. Glacial acetic acid (99.9%) was procured from Sisco Research Laboratory Pvt. Ltd., India. sodium thiosulfate (99%–100%) was supplied by Thermo Fisher Scientific Inc., and Supelco 37-component FAME mix, (CRM) purchased from Merck. All chemicals are analytical grade reagents.

### 2.3 Thermal treatment process

The heating temperature and duration (1, 2, 4 h) were decided based on the survey of street vendor stalls. Continuous 4 h exposure was designed to simulate the thermal stress experienced by oil in a single day, commonly practiced at street vendor stalls, thus representing the real-world frying conditions. The composite sample of each oil was subjected to controlled thermal treatment. For each composite oil, 100 mL was transferred into open stainless-steel containers to ensure uniform heat exposure. The samples were heated on a calibrated hotplate at a temperature of 230 ± 5 °C for durations of 1, 2, and 4 h. For each heating duration, three independent batch of composite oil was heated separately, and each was measured once. The reported triplicate values are the samples measured from the three independently heated batches (*n* = 3). Following the heat treatment, samples were cooled and transferred into amber colored, airtight containers before stored at −20 °C in the dark to minimize the further exposure to oxygen and light. Analysis was carried out within 2 days of storage and each sample used for analysis was thawed only once. While these precautions minimize oxidative changes during storage, residual oxidation during the freezing period cannot be entirely excluded and is acknowledged as a minor limitation of the study. Freezing preserves the overall quality of oils for up to 1 year, with characteristics closely resembling those of freshly produced oil over short term storage (29).

### 2.4 Analysis of fatty acid composition

Fatty acid profiling was performed in accordance with the AOAC (30) method, with minor modifications to enhance analytical consistency. A 40 *μ*L of each oil sample was mixed with 0.7 mL of 10 N KOH and 5.3 mL of methanol containing 0.05% butylated hydroxytoluene (BHT) as an antioxidant. The mixture was incubated in a water bath (JULABO GmbH, SW22, Germany) at 55 °C for 1 h 30 min. Thereafter, 0.58 mL of 24 N sulfuric acid (H₂SO₄) was added using 1 mL micro pipette (Eppendorf, Germany), and the mixture was transferred back into the water bath for an additional 1 h and 30 min. After cooling, 3 mL of n-hexane was added, and the mixture was centrifuged using Labortechnik Z366K centrifuge (Hermle Labortechnik GmbH, Wehingen, Germany) equipped with a swing-out rotor model 221.61 (radius 16.7 cm, 4 × 55.7 mL capacity) at ~743 × g for 3 min to separate the organic phase. The upper hexane layer was carefully transferred into a clean tube containing 0.1 g of anhydrous sodium sulphate (Na₂SO₄) to remove residual moisture. A portion of the dried extract was evaporated under nitrogen gas and reconstituted with 1 mL of dichloromethane. The final solution along with standard vials were transferred into gas chromatography (GC) autosampler for injection. Heptadecanoic acid (C17:0) was added to each sample as an internal standard for quantification. The analysis was carried out using gas chromatography (Agilent Technologies Inc., 7890A, USA) coupled with a flame ionization detector using a column namely CP-Sill 88 (100 m length, 0.25 mm diameter and 0.20 *μ*m film thickness). The sample (1 *μ*L) was injected in split mode with split ratio of 50:1, and hydrogen was used as a carrier gas with a constant total inlet flow rate of 40 mL/min for 45 min run time. The oven temperature was initially set at 120 °C, held for 0 min and then ramped to 200 °C at a rate of 4 °C/min and maintained for 5 min. The second ramp temperature was then increased at a rate of 4 °C/min to 240 °C, and the temperature was maintained for 10 min. The flame ionization detector (FID) temperature was set to 285 °C and injector was maintained at 225 °C. The peak was identified based on retention-time matching against reference standard. Selectivity was verified using 37 component FAME standard mix, conforming clear peak resolution and no observed interference in the vegetable oils. The method validation was demonstrated strong linearity with regression coefficients above 0.99 and confirming the accuracy with average recovery of 95%. The final results were expressed as FAME%.

### 2.5 Determination of peroxide value

The peroxide value (PV) was determined following the AOCS (30) method, with slight modifications. Approximately 3 g of oil sample was weighed in a 250 mL conical flask and dissolved in 30 mL of solvent mixture containing 3:2 ratio of acetic acid and chloroform. To the solution, 0.5 mL of a saturated aqueous potassium iodide (10 g/6 mL ultrapure water) solution was added. Then, the flask was allowed to stand for 1 min. After that, 30 mL of water was added, and the solution was titrated with 0.1 N sodium thiosulfate with constant agitation until the yellow colour of iodine had almost disappeared. About 0.5 mL of starch indicator solution (1% w/v in ultrapure water) was added, and titration was continued slowly until the blue colour disappeared. A blank titration was also performed, and the PV was derived from the following Equation 1:


$$\begin{array}{l} \text{Peroxide value}(\text{milli equivalents of}\frac{O_{2}}{\mathrm{kg}}\text{of oil})\\ =\frac{\begin{array}{l} \left(\text{Sample titre}-\text{Blank titre}\right)\\ \times \text{Normality of sodium thiosulphate solution}\times 1000 \end{array}}{\text{Weight of oil taken}\;(g)} \end{array}$$ (1)

### 2.6 Determination saponification value

The saponification value (SV) was analyzed following the method of Khaled et al. (31), with slight modifications. One gram of sample was weighed in a 250 mL conical flask with a ground-glass joint. To the sample, 25 mL of alcoholic KOH solution was added. The sample flask was connected to the air condenser. Then the sample was refluxed on a water bath for 30–45 min. The reaction mixture was steadily boiled until a homogeneous, clear solution was formed. Subsequently, the flask and condenser were allowed to cool to ambient temperature. The interior surface of the condenser was washed with 10 mL of ethanol. Followed by the addition of a few drops of phenolphthalein indicator before being titrated with 0.5 N HCl. A blank was performed alongside. Then the saponification value was calculated using the following Equation 2:


$$\begin{array}{l} \text{Saponification value}\;(\mathrm{mg}\;\text{of}\frac{\mathrm{KOH}}{g}\text{oil})\\ =\frac{(\text{Blank titre}-\text{Sample titre})\times \text{Normality of}\;\mathrm{HCl}\times 56.01}{\text{Weight of sample}(g)} \end{array}$$ (2)

### 2.7 Determination of acid value

The acid value (AV) was analysed following the method of Ashong et al. (32). Briefly, about 2–5 g of oil was weighed in 250 mL of conical flask, diluted with 25 mL of 95% ethanol and diethyl ether mixer (1:1 ratio), and heated to dissolve the oil. Then, two drops of phenolphthalein indicator added, and titrated with 0.1 N KOH till a pale pink colour appeared and remained consistent for 30 s. The acid value was calculated using the following Equation 3:


$$\begin{array}{l} \text{Acid value}\;(\mathrm{mg}\frac{\mathrm{KOH}}{g}\text{oil})\\ =\frac{\mathrm{ml}\;\text{of}\;\mathrm{KOH}\;\text{used}\;\times \text{Normality of}\;\mathrm{KOH}\;\times 56.01}{\text{Weight of sample}(g)} \end{array}$$ (3)

### 2.8 Determination of free fatty acid

The percentage of free fatty acid (FFA) was calculated as oleic acid by following the Ashong et al. (32) method. Approximately 2–5 g of the oil was weighed into the 250 mL of conical flask. Then, the sample was mixed with an alcoholic diethyl ether mixture (1:1 ratio, v/v), and warmed using hotplate (BAOSHISHAN, China). The sample was stirred to obtain a homogeneous mixture. Then, two drops of phenolphthalein indicator added, and titrated with 0.1 N KOH until a fair pink colour endpoint was obtained. The FFA was calculated using the following Equation 4:


$$\text{Free fatty acid}\;(\%\text{oleic acid})=\frac{\begin{array}{l} 28.2\times \text{Normality of}\;\mathrm{KOH}\\ \times \mathrm{ml}\;\text{of}\;\mathrm{KOH}\;\text{used} \end{array}}{\text{Weight of sample}(g)}$$ (4)

### 2.9 Determination of iodine value

The iodine value (IV) of oils was analysed by Wijs’s method, described by IS Protocol (1976), with slight modifications. Briefly, 0.5 g of the sample was mixed with 25 mL of chloroform. The mixture was further diluted with 25 mL of Wijs’s solution (10 mL of iodine monochloride in 2 litres of glacial acetic acid). The combination was allowed to stand for 1 h in the dark. After that, about 15 mL of KI (10%) and 100 mL of water was added to the mixture. The solution was titrated with 0.1 N sodium thiosulfate solution until straw-yellow colour appeared. Then, 1 mL of starch indicator was added and titrated till the colourless endpoint was obtained. A study of blank was performed alongside. The iodine value was calculated using the Equation 5:


$$\begin{array}{l} \text{Iodine value}\;(g\frac{I2}{100\;g}\text{oil})\\ =\frac{\begin{array}{l} \left(\text{Blank titre value}-\text{Sample titre value}\right)\\ \times \text{Normality of sodium thiosulfate}\times 12.69 \end{array}}{\text{Weight of oil taken}\;(g)} \end{array}$$ (5)

### 2.10 Determination of conjugated diene and triene

The conjugated diene (CD) and conjugated triene (CT) were determined by the spectrometric method following Karaogul et al. (33) method. The 0.01–0.05 g of oil sample was diluted with iso-octane. Then, absorbance was noted at 232 and 268 nm for CD and CT, respectively, by using UV spectrophotometer (UV-Plus, Motras Scientific, made in India). CD and CT were calculated using the following Equation 6:


$$E_{1\mathrm{cm}}^{1\%}=\frac{A}{C\times L}$$ (6)

Where, E = Extinction value, A = Absorbance measured at 232 (CD) or 268 (CT) nm, C = Concentration of the lipid in g/100 mL, and L = Path length of the cuvette.

### 2.11 Colour determination

The colour measurement (L*, Lightness) of edible oils was performed using a Hunter Lab Colour Flex EZ spectrometer (made in the USA). Prior to analysis, the instrument was calibrated with certified white and black standard tiles to establish 100 and 0% reflectance baselines, respectively. The calibration was verified against the manufacturer provided values to ensure accuracy within ±02 units. The oil sample was placed in a quartz cuvette and analyzed in transmittance mode, with each measurement replicated three times. Recalibration was performed in between the oil samples to get accuracy.

### 2.12 Determination refractive index

The refractive index (RI) of oil was determined using a digital refractometer (RX5000i, ATAGO, Japan). Firstly, the refractometer was calibrated with distilled water at laboratory temperature, then the prism was dried and cleaned. A drop of oil was placed on the prism using a pipette, and the cover plate was closed to ensure even distribution. The refractive index was read directly from the digital display after it stabilized. Multiple measurements were taken, averaged for accuracy and then the prism was cleaned between each trial to prevent contamination. The RI measurement was carried out at 40 °C. The final refractive index values were reported as the mean±standard deviation (SD).

### 2.13 Data analysis

All samples were measured in triplicate, corresponding to the three independently heated batches of the composite oil at each time point. This replication reflects independent repetition of the heat treatment of composite oil rather than independent sample of commercial brand or markets. Results are expressed as mean±SD. The fixed-effect two-way analysis of variance (ANOVA) was fitted separately for each response variable, according to the model, Yᵢⱼₖ = *μ* + *α*ᵢ + *β*ⱼ + (*αβ*)ᵢⱼ + εᵢⱼₖ, where *μ* is the grand mean, *α*ᵢ is the effect of oil type (i = 1–12), *β*ⱼ is the effect of heating time (*j* = 1–4), (*αβ*)ᵢⱼ is the oil type × heating time interaction, and εᵢⱼₖ is the independently distributed residual error for replicate k (k = 1–3). This analysis was performed to determine whether oil type, heating duration, and their interaction have statistically significant effect on each measured parameter. Additionally, student’s t-test was conducted to determine whether there was statistically significant difference between the CP and RF type of same oil, using IBM SPSS Statistics software, version 23.0 (IBM Corp., USA). Prior to perform ANOVA, the assumption of homogeneity of variances was tested using Levene’s test, where homogeneity of variances was satisfied, Tukey’s HSD test (*p* ≥ 0.05) was applied for *post hoc* comparisons and where the assumption of homogeneity of variances was violated, the Games-Howell (*p* < 0.05) *post hoc* test was used. Because twelve dependent variables were tested, a Bonferroni-adjusted significance threshold (*α* = 0.05/12 = 0.0042) was also applied to evaluate the robustness of the oil × time interaction across outcomes. All interaction effects remained significant under this threshold except for RI (*p* = 0.028), for which interaction effect interpreted with caution; the corresponding 95% confidence interval for partial *η*^2^ (0.00–0.37) likewise approached zero, consistent with a comparatively fragile interaction effect for this parameter relative to the others. The principle component analysis (PCA) was performed using the FactoMineR and ggplot2packages in R software. The FFA content was excluded from the PCA input variables due to strong collinearity with AV. Because the four fatty acid composition variables (TSFA, TMUFA, TPUFA, TTFA), constitute closed compositional data summing to approximately 100%, they were subjected to centered log-ratio (CLR) transformed prior to PCA to remove the linear dependency inherent to closed data and avoid spurious correlations arising from data closure. Zero values (present only for TTFA in unheated samples) were replaced with a small pseudo-count prior to transformation. The remaining physicochemical parameters (PV, AV, IV, SV, CD, CT, RI, L*) were retained on their original scale and Z standardized alongside the CLR transformed variables before PCA. Principal components with eigenvalues greater than 1 were examined, and biplot integrating sample score scatter points and variable loading vectors was constructed to visualize sample clustering and parameter correlations.

## 3 Results

### 3.1 Effect of thermal treatment on fatty acid composition of cold pressed and refined oils

The effect of thermal treatment on fatty acid composition of CP and RF oils is presented in Table 1. Among the oils represented in the both processing types (groundnut, sunflower, rice bran, and palm oil), CP oils exhibited higher retention of nutritional quality during shorter period of heating (1 h), but greater loss under continuous exposure (4 h) of thermal treatment than the RF oils. In total saturated fatty acid (TSFA), CP coconut oil demonstrated exceptional thermal stability, only increased from 90.12 ± 0.17 to 99.01 ± 0.01%, in contrast soybean (RF), mustard (CP), and sesame oil (CP) showed a sharp rise of 25.11 ± 0.17, 10.48 ± 0.04 and 23.2 ± 0.14%, respectively after 4 h. Total mono unsaturated fatty acid (TMUFA) composition exhibited oil-specific variations. In most oils, including groundnut (CP & RF), sunflower (CP & RF), rice bran (CP & RF), soybean (RF) and mustard oil (CP) exhibited an increase in TMUFA, largely attributable to total poly unsaturated fatty acid (TPUFA) degradation and rearrangements. Conversely, palm (CP, 41.31 ± 0.01 to 40.64 ± 0.06; RF, 44.99 ± 0.08 to 42.60 ± 0.04%) and CP coconut (7.71 ± 0.13 to 0.62 ± 0.03%) demonstrated marked TMUFA losses after 4 h of heating. TPUFA retention varied markedly among oils. After 1 h, reduction of TPUFA was less in CP rice bran (33.10 ± 0.01 to 31.35 ± 0.09) and RF sunflower oil (59.44 ± 0.16 to 56.19 ± 0.06%), while, RF palm (10.74 ± 0.01 to 8.57 ± 0.63%) and CP coconut oil (2.17 ± 0.05 to 0.86 ± 0.01%) showed the highest. After 2 h, CP rice bran oil remained relatively stable with 31.06 ± 0.02% followed by RF sunflower oil with 55.02 ± 0.01%, whereas CP coconut oil (0.39 ± 0.03%) and palm oil (8.76 ± 0.01%) declined sharply. After 4 h, RF sunflower oil, followed by CP sunflower and rice bran oils exhibited minimal change, but CP coconut oil showed maximum TPUFA loss. The total trans fatty acid (TTFA) was negligible in non-heated CP oils, but increased progressively with heating. The RF soybean oil showed a sharp rise (0.76 ± 0.08 to 5.88 ± 0.04%), followed by CP sesame oil (0.16 ± 0.01 to 4.05 ± 0.01%). CP coconut oil recorded the lowest TTFA with (0 to 0.38 ± 0.03%) after 4 h of thermal exposure.

**Table 1:** Effect of thermal treatment on fatty acid composition of cold-pressed and refined oils.

Oil name	Oil type	Saturated fatty acid			
		0 h	1 h	2 h	4 h
Groundnut oil	Cold pressed	22.75 ± 0.40^a,4,x^	24.19 ± 0.10^b,5,x^	27.05 ± 0.22^c,4,x^	28.50 ± 0.70^d,5,x^
	Refined	20.39 ± 0.02^a,6,y^	21.83 ± 0.04^b,7,y^	23.54 ± 0.05^c,7,y^	24.77 ± 0.08^d,8,y^
Sunflower oil	Cold pressed	10.85 ± 0.05^a,9,x^	12.21 ± 0.02^b,10,x^	12.77 ± 0.05^c,10,x^	13.49 ± 0.10^d,10,x^
	Refined	10.78 ± 0.08^a,9,x^	12.13 ± 0.06^b,10,x^	12.47 ± 0.01^c,11,y^	12.74 ± 0.02^d,11,y^
Rice bran oil	Cold pressed	22.80 ± 0.04^a,4,x^	23.82 ± 0.15^b,6,x^	23.81 ± 0.03^b,6,x^	26.85 ± 0.16^c,6,x^
	Refined	22.50 ± 0.13^a,5,y^	24.76 ± 0.02^b,4,y^	24.79 ± 0.38^b,5,y^	29.06 ± 0.15^c,4,y^
Palm oil	Cold pressed	46.53 ± 0.07^a,2,x^	47.56 ± 0.04^b,2,x^	49.95 ± 0.03^c,2,x^	51.82 ± 0.01^d,2,x^
	Refined	43.93 ± 0.08^a,3,y^	46.33 ± 0.41^b,3,y^	46.95 ± 0.01^c,3,y^	51.06 ± 0.10^d,3,y^
Mustard oil	Cold pressed	7.28 ± 0.05^a,10^	7.98 ± 0.07^c,11^	7.84 ± 0.02^b,12^	10.48 ± 0.04^d,12^
Sesame oil	Cold pressed	16.85 ± 0.06^a,7^	18.09 ± 0.06^b,9^	18.97 ± 0.04^c,9^	23.2 ± 0.14^d,9^
Soybean oil	Refined	16.15 ± 0.08^a,8^	18.45 ± 0.02^b,8^	19.01 ± 0.02^c,8^	25.11 ± 0.17^d,7^
Coconut oil	Cold pressed	90.12 ± 0.17^a,1^	92.88 ± 0.07^b,1^	94.40 ± 0.05^c,1^	99.01 ± 0.01^d,1^
Monounsaturated fatty acid					
Groundnut oil	Cold pressed	44.43 ± 0.15^a,4,x^	45.53 ± 0.04^b,3,x^	46.75 ± 0.07^c,3,x^	46.62 ± 0.38^c,4,x^
	Refined	46.53 ± 0.10^a,2,y^	47.98 ± 0.03^b,2,y^	49.27 ± 0.01^c,2,y^	49.79 ± 0.02^d,2,y^
Sunflower oil	Cold pressed	28.33 ± 0.08^a,10,x^	30.20 ± 0.03^b,10,x^	30.87 ± 0.02^c,10,x^	31.66 ± 0.14^d,9,x^
	Refined	28.85 ± 0.09^a,9,y^	30.70 ± 0.01^b,9,x^	31.23 ± 0.01^c,9,y^	31.47 ± 0.10^d,10,x^
Rice bran oil	Cold pressed	43.10 ± 0.14^a,5,x^	43.72 ± 0.08^b,5,x^	43.99 ± 0.03^c,6,x^	45.92 ± 0.10^d,6,x^
	Refined	41.49 ± 0.18^a,7,y^	42.54 ± 0.04^b,7,y^	43.45 ± 0.28^c,7,y^	46.02 ± 0.11^d,5,x^
Palm oil	Cold pressed	41.31 ± 0.01^b,8,x^	41.49 ± 0.05^a,8,x^	41.29 ± 0.02^b,8,x^	40.64 ± 0.06^c,8,x^
	Refined	44.99 ± 0.08^a,3,y^	44.27 ± 0.22^b,4,y^	44.44 ± 0.02^b,4,y^	42.60 ± 0.04^c,7,y^
Mustard oil	Cold pressed	62.09 ± 0.20^a,1^	63.01 ± 0.37^b,1^	64.05 ± 0.03^c,1^	69.82 ± 0.01^d,1^
Sesame oil	Cold pressed	42.16 ± 0.01^a,6^	43.46 ± 0.02^b,6^	44.08 ± 0.01^c,5^	47.20 ± 0.04^d,3^
Soybean oil	Refined	21.89 ± 0.12^a,11^	24.26 ± 0.02^b,11^	24.28 ± 0.04^b,11^	28.59 ± 0.09^c,11^
Coconut oil	Cold pressed	7.71 ± 0.13^d,12^	6.09 ± 0.07^c,12^	4.98 ± 0.02^b,12^	0.62 ± 0.03^a,12^
Polyunsaturated fatty acid					
Groundnut oil	Cold pressed	32.82 ± 0.25^d,7,x^	30.28 ± 0.05^c,7,x^	24.72 ± 0.16^b,9,x^	21.02 ± 0.30^a,8,x^
	Refined	32.17 ± 0.04^d,5-7,y^	29.12 ± 0.01^c,8,y^	25.27 ± 0.09^b,8,y^	21.92 ± 0.02^a,6,y^
Sunflower oil	Cold pressed	60.82 ± 0.13^d,2,x^	56.63 ± 0.01^c,1,x^	55.18 ± 0.07^b,2,x^	53.23 ± 0.21^a,2,x^
	Refined	59.44 ± 0.16^d,3,y^	56.19 ± 0.06^c,2,x^	55.02 ± 0.01^b,3,y^	54.37 ± 0.06^a,1,x^
Rice bran oil	Cold pressed	33.10 ± 0.01^d,6,x^	31.35 ± 0.09^c,5,x^	31.06 ± 0.02^b,5,x^	25.11 ± 0.02^a,5,x^
	Refined	34.79 ± 0.27^c,5,y^	30.73 ± 0.03^b,6,y^	30.31 ± 0.70^b,6,x^	21.53 ± 0.08^a,7,x^
Palm oil	Cold pressed	12.16 ± 0.07^d,9,x^	10.83 ± 0.01^c,10,x^	8.76 ± 0.01^b,10,x^	7.14 ± 0.05^a,10,x^
	Refined	10.74 ± 0.01^c,11,y^	8.57 ± 0.63^b,11,y^	8.07 ± 0.01^b,11,y^	5.17 ± 0.01^a,11,y^
Mustard oil	Cold pressed	30.64 ± 0.15^d,8^	28.35 ± 0.45^c,9^	27.13 ± 0.03^b,7^	17.31 ± 0.04^a,9^
Sesame oil	Cold pressed	40.84 ± 0.06^d,4^	38.01 ± 0.06^c,4^	36.24 ± 0.04^b,4^	25.55 ± 0.12^a,4^
Soybean oil	Refined	61.20 ± 0.11^d,1^	56.05 ± 0.02^c,3^	55.28 ± 0.03^b,1^	40.65 ± 0.01^a,3^
Coconut oil	Cold pressed	2.17 ± 0.05^c,11^	0.86 ± 0.01^b,12^	0.39 ± 0.03^a,12^	0 ± 0
Trans fatty acid					
Groundnut oil	Cold pressed	0 ± 0	0 ± 0	1.49 ± 0.01^a,2,x^	3.86 ± 0.02^b,3,x^
	Refined	0.92 ± 0.04^a,2,x^	1.08 ± 0.07^b,3,x^	1.92 ± 0.05^c,1,y^	3.52 ± 0.07^d,4,y^
Sunflower oil	Cold pressed	0 ± 0	0.97 ± 0.03^a,3,x^	1.18 ± 0.01^b,3,x^	1.62 ± 0.03^c,8,x^
	Refined	0.93 ± 0.03^a,3,x^	0.98 ± 0.04^a,2,x^	1.28 ± 0.01^b,3,y^	1.42 ± 0.02^c,9,y^
Rice bran oil	Cold pressed	0.99 ± 0.10^a,2,x^	1.11 ± 0.01^b,2,x^	1.13 ± 0.03^b,3,x^	2.11 ± 0.05^c,7,x^
	Refined	1.22 ± 0.04^a,1,y^	1.26 ± 0.04^a,1,y^	1.45 ± 0.04^b,2,y^	3.40 ± 0.03^c,5,y^
Palm oil	Cold pressed	0 ± 0	0.12 ± 0.01^a,8,x^	0.29 ± 0.01^b,7,x^	0.40 ± 0.01^c,11,x^
	Refined	0.34 ± 0.01^a,5,x^	0.83 ± 0.01^c,4,y^	0.54 ± 0.03^b,6,y^	1.16 ± 0.05^d,10,y^
Mustard oil	Cold pressed	0 ± 0	0.67 ± 0.01^a,5^	0.97 ± 0.01^b,4^	2.39 ± 0.01^c,6^
Sesame oil	Cold pressed	0.16 ± 0.01^a,6^	0.45 ± 0.02^b,6^	0.71 ± 0.01^c,5^	4.05 ± 0.01^d,2^
Soybean oil	Refined	0.76 ± 0.08^a,4^	1.24 ± 0.06^b,2^	1.43 ± 0.03^c,2^	5.88 ± 0.04^d,1^
Coconut oil	Cold pressed	0 ± 0	0.17 ± 0.01^a,7^	0.23 ± 0.01^b,8^	0.38 ± 0.03^c,11^

Values are mean±SD (*n* = 3). Values in same row with different superscript letters (a–d) across heating durations differ significantly and values in same column with different superscript numbers (1–12) across oils differ significantly (two-way ANOVA with Tukey’s HSD post hoc test where Levene’s test *p* ≥ 0.05; Games-Howell post hoc test where Levene’s test *p* < 0.05). Values in same column with different superscript letters (x,y) between cold-pressed and refined type of the same oil differ significantly (Student’s *t*-test, *p* ≤ 0.05).

### 3.2 Effect of thermal treatment on peroxide value of cold pressed and refined oils

The peroxide value (PV), was determined for CP and RF oils under thermal exposure, as presented in Figure 1 (expressed as active oxygen per 1 kg of oil). In non-heated oil sample, all oils were within codex limits (up to10 and 15 mEq O₂/kg for refined and cold pressed oil, respectively), except for CP palm oil (15.39 ± 0.01 mEq O_2_/kg). Groundnut (CP & RF) and coconut oil (CP) surpassed the standard PV after 1 h of heating, followed by decline at 2 and 4 h. Sunflower oil (RF) exceeded the limit after 4 h (15.81 ± 0.02 mEq O_2_/kg), whereas CP remained within the codex limit (7.95 ± 1.85 mEq O_2_/kg). Rice bran oil (CP & RF) showed continuous decline, reached its lowest PV (0.01 ± 0.01 mEq O_2_/kg) after 2 h and continued the same in further heating. Similarly, CP mustard oil also exhibited a steady decline (7.68 ± 0.01 to 3.19 ± 0.01 mEq O_2_/kg oil) throughout the thermal process. Non-heated sesame oil (CP) exhibited the lowest PV (1.60 ± 0.01 mEq O_2_/kg). Soybean (RF), palm (RF) and coconut oil (CP) showed initial PV of 3.85 ± 0.01 mEq O_2_/kg; Upon heating for 1 h, three oils displayed sharp rise, CP coconut oil reached highest PV with 28.62 ± 0.07 mEq O_2_/kg, before declining with continuous thermal exposure. Pronounced variation was observed between CP and RF palm oil. The PV in CP palm oil was declined progressively (15.39 ± 0.01 to 3.17 ± 0.01 mEq O_2_/kg), whereas RF increased initially (3.85 ± 0.01 to 9.53 ± 0.01 mEq O_2_/kg) at 2 h, before decreasing to 6.36 ± 0.01 mEq O_2_/kg, after 4 h of thermal exposure.

**Figure 1:**
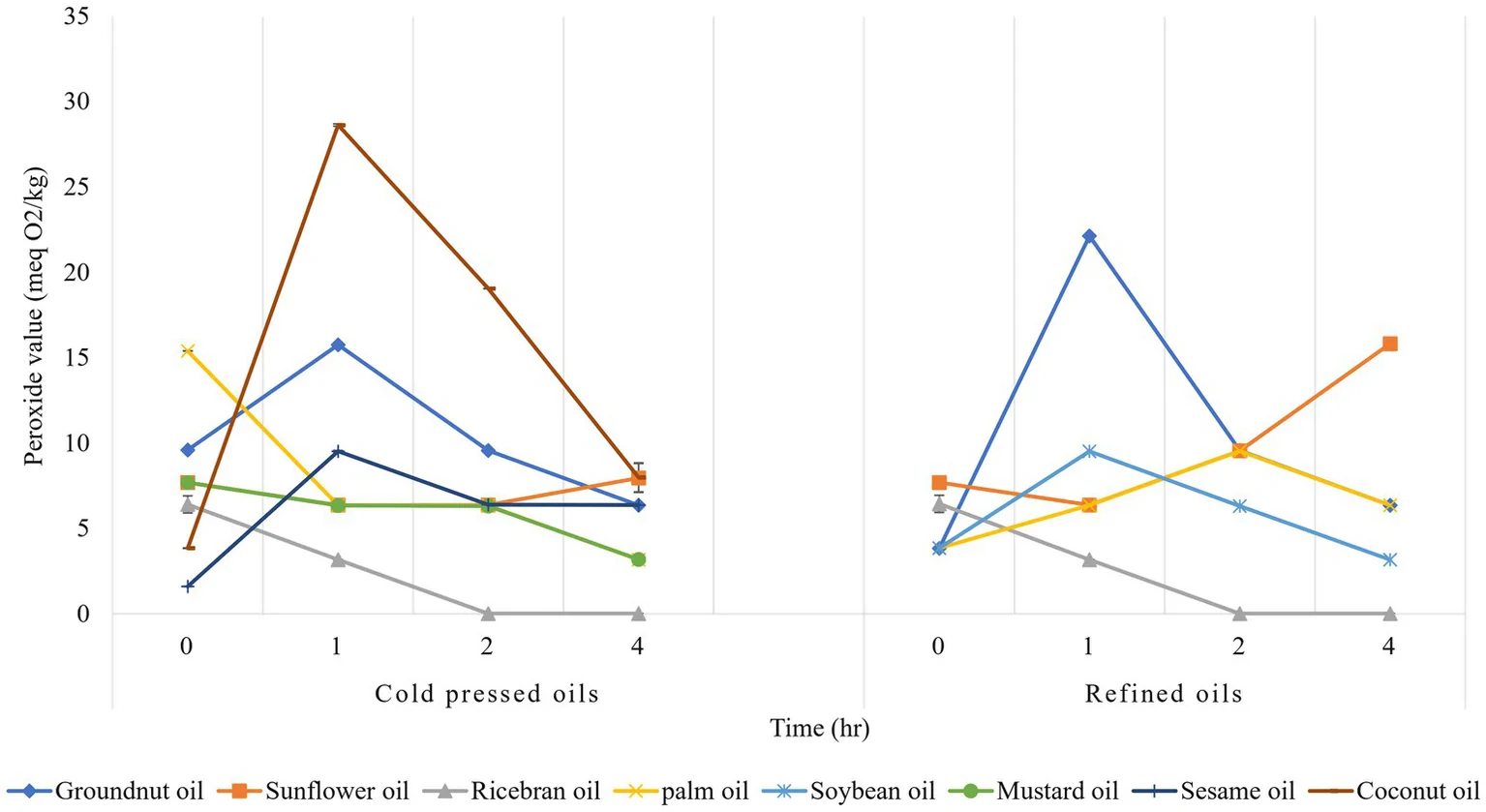
Effect of thermal treatment on the peroxide value of cold-pressed and refined oils.

### 3.3 Effect of thermal treatment on saponification value of cold pressed and refined oils

The data represented in Figure 2 demonstrate the saponification value (SV) of the CP and RF oils under thermal treatment, highlighting variation in molecular composition among different oil types. Sunflower oil (CP & RF) showed progressive increase in SV during the first 2 h of heating (186 ± 0.32 to 224 ± 0.99 and 190 ± 0.83 to 236 ± 0.86 mg KOH/g, respectively), followed by decline with prolonged heating. In contrast, CP mustard and sesame oil demonstrated an initial reduction in SV during the first 2 h, which subsequently increased upon continuous heating. CP coconut oil displayed consistent upward trend in SV throughout the entire heating duration (269 ± 0.53 to 321 ± 0.68 mg KOH/g). Groundnut (CP & RF), rice bran (CP), palm (CP & RF), and soybean oil (RF) showed fluctuated results, an initial rise at 1 h, decline at 2 h and renewed increase at 4 h. Rice bran oil demonstrated a notable difference between the CP and RF. Rice bran oil (RF) showed a marked decline after 1 h (191 ± 1.16 to 110 ± 0.77 mg KOH/g), followed by sharp increase at 2 h (142 ± 0.82 mg KOH/g) and 4 h (261 ± 0.90 mg KOH/g) of thermal exposure.

**Figure 2:**
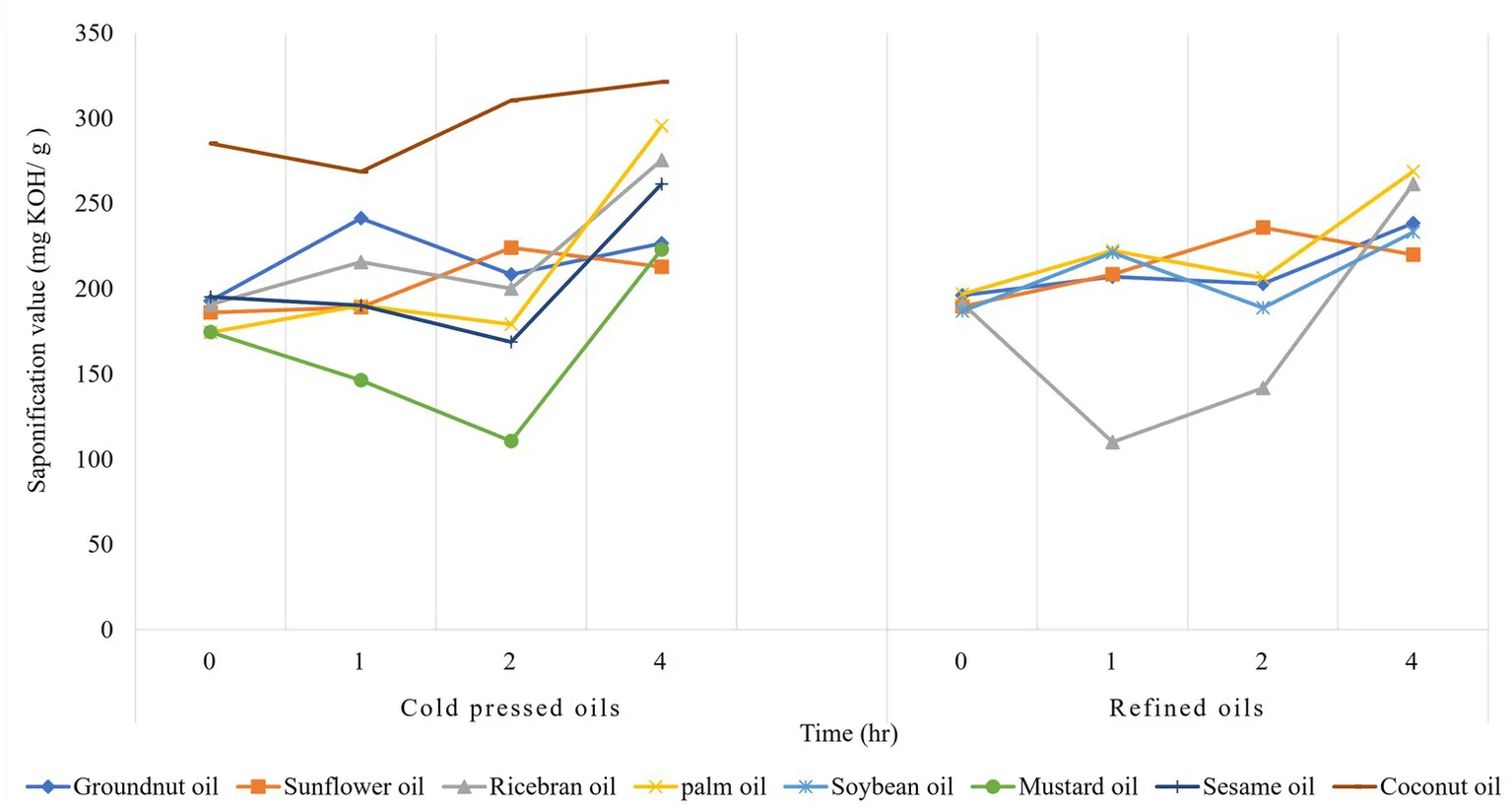
Effect of thermal treatment on saponification value of cold-pressed and refined oils.

### 3.4 Effect of thermal treatment on acid value and free fatty acid of cold pressed and refined oils

Table 2 represents the results of free fatty acid value (FFA, expressed as oleic acid %) and acid value (AV, mg KOH/g) of thermally exposed CP and RF oils. In the present study, FFA levels of all oils declined progressively with heating. Among non-heated oils, sunflower (CP) and soybean (RF) exhibited the lowest FFA (0.89 ± 0.05%), while CP palm and sesame oil showed the highest value 8.26 and 5.68%, respectively. After 1 h of heating, CP groundnut (1.40 ± 0.01%), rice bran (1.11 ± 0.01%) and palm oil (6.27 ± 0.01%) showed higher FFA than their RF types with 0.42 ± 0.01, 0.55 ± 0.01 and 0.51 ± 0.08%, respectively. Rice bran oil demonstrated sharp decline in both CP and RF types, reaching negligible levels after 1 to 2 h of heating (1.55 ± 0.05 to 0.09 ± 0.01 and 1.42 ± 0.05–0.09 ± 0.01%, respectively) and remained minimal thereafter. CP sesame and coconut oil was reached their lowest FFA after 4 h, declining from 5.68 ± 0.14 to 0.28 ± 0.01 and 1.55 ± 0.27 to 0.28 ± 0.01%, respectively. Acid value (AV) mirrored the FFA trend. Among non-heated CP oils, palm oil recorded the highest AV (16.44 ± 0.13 mg KOH/g), followed by sesame (11.3 ± 0.27), groundnut (4.24 ± 0.82), and coconut (3.08 ± 0.54 mg KOH/g). Overall, all CP oils exhibited higher AV than RF oils, while both CP and RF showed a progressive decline in AV during heating.

**Table 2:** Effect of thermal treatment on the free fatty acid and acid value of cold pressed and refined oils.

Oil name	Oil type	Free fatty acid (% oleic acid)				Acid value (mg KOH/g oil)			
		0 h	1 h	2 h	4 h	0 h	1 h	2 h	4 h
Groundnut oil	Cold pressed	2.13 ± 0.41^c,3,x^	1.40 ± 0.01^b,3,x^	0.34 ± 0.05^a,2,x^	0.28 ± 0.01^a,2,x^	4.24 ± 0.82^c,3,x^	2.78 ± 0.01^b,3,x^	0.62 ± 0.11^a,2,x^	0.55 ± 0.01^a,2,x^
	Refined	0.92 ± 0.09^c,4,x^	0.42 ± 0.01^b,6,y^	0.37 ± 0.01^b,2,x^	0.28 ± 0.01^a,3,x^	1.84 ± 0.19^c,4,y^	0.84 ± 0.01^b,6,y^	0.74 ± 0.01^b,2,x^	0.55 ± 0.01^a,3,x^
Sunflower oil	Cold pressed	0.89 ± 0.05^b,4,x^	0.28 ± 0.01^a,7,x^	0.28 ± 0.01^a,3,x^	0.34 ± 0.05^a,1,x^	1.78 ± 0.10^b,4,x^	0.55 ± 0.01^a,7,x^	0.56 ± 0.01^a,3,x^	0.68 ± 0.11^a,1,x^
	Refined	0.93 ± 0.01^d,4,x^	0.28 ± 0.01^a,7,x^	0.37 ± 0.01^b,2,y^	0.37 ± 0.01^b,1,x^	1.84 ± 0.01^c,4,x^	0.56 ± 0.01^a,7,x^	0.74 ± 0.01^b,2,y^	0.73 ± 0.01^b,1,x^
Rice bran oil	Cold pressed	1.55 ± 0.05^c,3,x^	1.11 ± 0.01^b,4,x^	0.09 ± 0.01^a,5,x^	0.09 ± 0.01^a,5,x^	3.08 ± 0.10^c,3,x^	2.20 ± 0.01^b,4,x^	0.19 ± 0.01^a,6,x^	0.18 ± 0.01^a,6,x^
	Refined	1.42 ± 0.05^c,3,y^	0.55 ± 0.01^b,5,y^	0.09 ± 0.01^a,5,x^	0.09 ± 0.01^a,5,x^	2.82 ± 0.11^c,3,y^	1.10 ± 0.01^b,5,y^	0.18 ± 0.01^a,6,x^	0.18 ± 0.01^a,6,x^
Palm oil	Cold pressed	8.26 ± 0.07^d,1,x^	6.27 ± 0.01^c,1,x^	0.28 ± 0.01^b,4,x^	0.18 ± 0.01^a,4,x^	16.44 ± 0.13^d,1,x^	12.46 ± 0.03^c,1,x^	0.55 ± 0.01^b,4,x^	0.37 ± 0.01^a,4,x^
	Refined	1.32 ± 0.05^d,3,y^	0.51 ± 0.08^c,5,y^	0.37 ± 0.01^b,2,y^	0.28 ± 0.01^a,2,y^	2.63 ± 0.10^d,3,y^	1.02 ± 0.16^c,5,y^	0.74 ± 0.01^b,2,y^	0.55 ± 0.01^a,2,y^
Mustard oil	Cold pressed	1.42 ± 0.05^c,3^	0.74 ± 0.08^b,4^	0.25 ± 0.05^a,2–4^	0.19 ± 0.01^a,4^	2.83 ± 0.11^c,3^	1.48 ± 0.16^b,4^	0.43 ± 0.11^a,2–5^	0.37 ± 0.01^a,4^
Sesame oil	Cold pressed	5.68 ± 0.14^c,2^	2.75 ± 0.02^b,2^	0.28 ± 0.01^a,3^	0.28 ± 0.01^a,2^	11.3 ± 0.27^c,2^	5.47 ± 0.05^b,2^	0.56 ± 0.01^a,3^	0.56 ± 0.01^a,2^
Soybean oil	Refined	0.89 ± 0.05^c,4^	0.27 ± 0.01^b,7^	0.28 ± 0.01^b,4^	0.18 ± 0.01^a,4^	1.78 ± 0.10^c,4^	0.55 ± 0.01^a,8^	0.55 ± 0.01^a,5^	0.37 ± 0.01^b,5^
Coconut oil	Cold pressed	1.55 ± 0.27^c,3^	0.78 ± 0.08^b,4^	0.71 ± 0.05^b,1^	0.28 ± 0.01^a,2^	3.08 ± 0.54^c,3^	1.56 ± 0.15^b,4^	1.41 ± 0.11^a,1^	0.56 ± 0.01^a,2^

Values are mean±SD (*n* = 3). Values in same row with different superscript letters (a–d) across heating durations differ significantly and values in same column with different superscript numbers (1–12) across oils differ significantly (two-way ANOVA with Tukey's HSD post hoc test where Levene’s test *p* ≥ 0.05; Games-Howell post hoc test where Levene’s test *p* < 0.05). Values in same column with different superscript letters (x,y) between cold-pressed and refined type of the same oil differ significantly (Student’s *t*-test, *p* ≤ 0.05).

### 3.5 Effect of thermal treatment on iodine value of cold pressed and refined oil

Figure 3 represents the iodine value (IV) of CP and RF oils under thermal treatment. Progressive decline in IV was observed across all oils as heating duration increased. Among fresh sample, RF sunflower oil exhibited the highest IV (138 ± 1.39 g I_2_/100 g), whereas CP coconut oil showed the lowest (18.74 ± 1.26 g I_2_/100 g). RF groundnut (103 ± 1.18), sunflower (138 ± 1.39) and rice bran oil (112 ± 1.42) exhibit higher IV than the CP oils (103 ± 1.18, 134 ± 0.85 and 109 ± 0.5 g I_2_/100 g, respectively). After 1 h of heating, no significant reduction was observed in CP groundnut and sunflower oils, whereas their RF types showed reduction of 5.57 and 6.68%, respectively. By 2 h, reduction was noted in RF groundnut, rice bran oil (CP & RF), mustard oil (CP), and soybean oil (RF), though these changes were not statistically significant. RF sunflower oil showed only minor alterations between 2 to 4 h (121.85 ± 1.61 to 121.49 ± 0.61 g I_2_/100 g). The most pronounced decline occurred in CP coconut oil and palm oil also showed substantial decrease in CP 30.63%, and RF oil 30.3% after 4 h of thermal exposure. In contrast, minimal reduction was observed in RF soybean (6.72%), CP rice bran (7.20%) and groundnut oil (CP, 8.82 and RF, 12.10%) and after 4 h of continuous thermal treatment.

**Figure 3:**
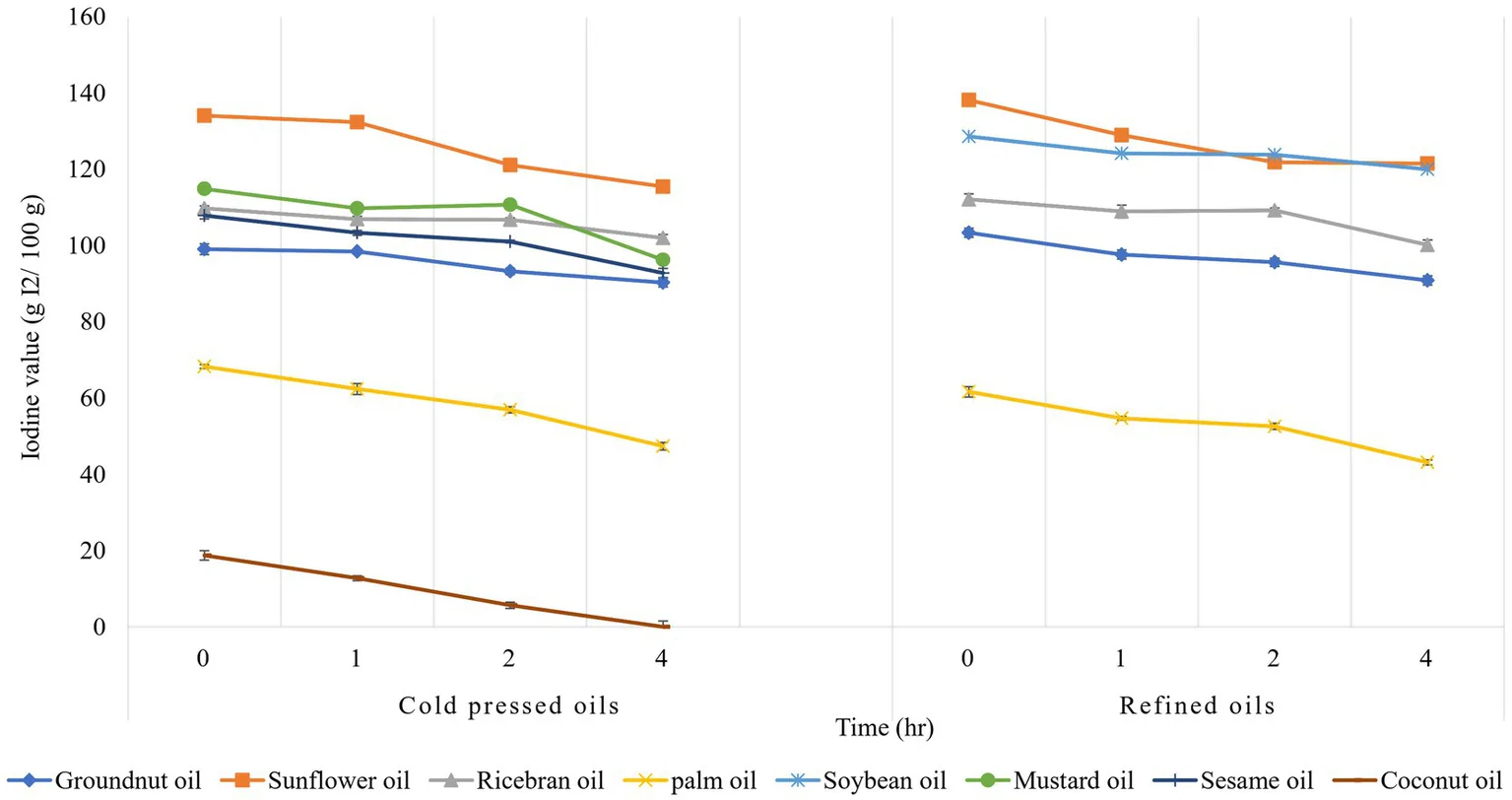
Effect of thermal treatment on the iodine value of cold-pressed and refined oils.

### 3.6 Effect of thermal treatment on conjugated diene and trienes of cold pressed and refined oils

In the present study, conjugated triene (CT) values remained lower than conjugated diene (CD) values (Table 3) across all oil samples. In groundnut oil (CP & RF), CD levels increased significantly during heating. The CP groundnut oil rose from 6.57 ± 0.4 to peak of 13.93 ± 0.65 after 1 h, while RF increased from 2.22 ± 0.27 to 11.44 ± 0.74 after 2 h, followed by a decline with continuous heating. Sunflower oil exhibited difference between CP and RF throughout the thermal process. After 1 h, CP sunflower oil exhibited lower CD formation (5.42 ± 0.18) compared to RF (10.92 ± 0.53). By 2 h, CD values increased sharply in both oils, reaching 17.51 ± 0.37 (CP) and 17.83 ± 0.32 (RF). Rice bran oil exhibited an initially declined in CD levels after 1 h (CP, 10.28 ± 0.18 to 5.32 ± 0.18; RF, 14.86 ± 0.42 to 7.89 ± 0.54), followed by a progressive increase during 2 to 4 h of heating, with CP consistently lower than RF. Palm oil exhibited variable CD trends. CP palm oil showed fluctuations, while RF palm oil declined initially and then increased gradually. After 1 h, CD values were lowest among all oils (CP, 2.85 ± 0.17; and RF, 2.57 ± 0.23). RF soybean oil produced 14.07 ± 0.36 of CD after 2 h. CP mustard, sesame, and coconut oils demonstrated a steady increase in CD values throughout the heating duration. Continuous heating resulted in elevated CT levels in CP and RF. The CP palm, mustard, and coconut oil exhibited an initial decline in CT concentration during the 1 h of heating, followed by a subsequent rise with continued thermal exposure.

**Table 3:** Effect of thermal treatment on the conjugated dienes and trienes of cold-pressed and refined oils.

Oil name	Oil type	Dienes (K_232_)				Trienes (K_268_)			
		0 h	1 h	2 h	4 h	0 h	1 h	2 h	4 h
Groundnut oil	Cold pressed	6.57 ± 0.40^a,3,x^	13.93 ± 0.65^d,1,x^	9.86 ± 0.30^c,4,x^	8.87 ± 0.55^b,4,x^	0.48 ± 0.02^a,x^	1.52 ± 0.17^b,5,x^	4.45 ± 0.18^c,3,x^	4.82 ± 0.25^d,3,x^
	Refined	2.22 ± 0.27^a,5,y^	9.93 ± 0.46^b,2,y^	11.44 ± 0.74^c,2,y^	9.21 ± 0.51^b,3,x^	2.40 ± 0.06^b,y^	3.87 ± 0.01^c,3,y^	4.71 ± 0.30^d,3,x^	6.30 ± 0.01^a,5,y^
Sunflower oil	Cold pressed	4.17 ± 0.02^a,4,x^	5.42 ± 0.18^b,4,x^	17.51 ± 0.37^d,1,x^	13.48 ± 0.79^c,2,x^	1.93 ± 0.23^a,x^	1.20 ± 0.01^a,6,x^	8.74 ± 0.90^b,1,x^	9.52 ± 0.27^b,1,x^
	Refined	5.39 ± 0.22^a,3,y^	10.92 ± 0.53^b,2,y^	17.83 ± 0.32^d,1,x^	16.53 ± 0.63^c,1,y^	0.80 ± 0.03^a,y^	6.27 ± 0.27^b,2,y^	9.64 ± 0.05^c,1,x^	10.53 ± 0.40^d,1,y^
Rice bran oil	Cold pressed	10.28 ± 0.18^d,2,x^	5.32 ± 0.18^a,4,x^	8.06 ± 0.52^b,5,x^	9.02 ± 0.73^c,4,x^	3.05 ± 0.05^a,x^	4.52 ± 0.14^b,3,x^	5.88 ± 0.38^c,2,x^	8.91 ± 0.44^d,2,x^
	Refined	14.86 ± 0.42^c,1,y^	7.89 ± 0.54^a,3,y^	12.05 ± 0.81^b,3,y^	12.42 ± 0.86^b,2,y^	3.93 ± 0.12^a,y^	7.62 ± 0.33^b,1,y^	9.99 ± 0.49^c,1,y^	11.25 ± 0.74^d,1,y^
Palm oil	Cold pressed	5.36 ± 0.48^b,3,x^	2.85 ± 0.17^a,5,x^	6.63 ± 0.64^b,5,x^	5.59 ± 0.22^b,5,x^	0.78 ± 0.01^b,x^	0.23 ± 0.02^a,8,x^	3.41 ± 0.17^c,4,x^	4.88 ± 0.31^d,3,x^
	Refined	2.96 ± 0.09^a,5,y^	2.57 ± 0.23^a,5,x^	5.19 ± 0.09^b,6,y^	6.79 ± 0.98^c,5,x^	0.44 ± 0.02^a,y^	0.66 ± 0.06^a,7,y^	2.11 ± 0.23^b,5,y^	4.35 ± 0.27^c,3,x^
Mustard oil	Cold pressed	3.78 ± 0.11^a,4^	3.53 ± 0.25^a,5^	7.76 ± 0.52^b,5^	9.55 ± 0.35^c,3^	4.92 ± 0.32^b^	3.67 ± 0.14^a,4^	6.69 ± 0.19^c,2^	9.98 ± 0.14^d,1^
Sesame oil	Cold pressed	3.86 ± 0.12^a,4^	5.06 ± 0.23^b^	8.03 ± 0.75^c,5^	11.05 ± 0.57^d,3^	0.23 ± 0.04^a^	2.31 ± 0.09^b,5^	4.56 ± 0.28^c,3^	9.17 ± 0.38^d,2^
Soybean oil	Refined	5.53 ± 0.05^a,3^	10.9 ± 0.28^c,2^	14.07 ± 0.36^d,2^	8.6 ± 0.55^b,4^	2.50 ± 0.20^a^	7.77 ± 0.33^b,1^	10.51 ± 0.38^c,1^	12.03 ± 0.61^d,1^
Coconut oil	Cold pressed	1.89 ± 0.17^a,6^	3.36 ± 0.45^b,5^	5.72 ± 0.71^c,6^	7.37 ± 0.42^d,4^	0.10 ± 0.02^b^	0.03 ± 0.01^a,9^	0.72 ± 0.06^c,6^	3.00 ± 0.03^d,4^

Values are mean±SD (*n* = 3) of specific extinction coefficients (dL g^−1^ cm^−1^). Values in same row with different superscript letters (a–d) across heating durations differ significantly and values in same column with different superscript numbers (1–12) across oils differ significantly (two-way ANOVA with Tukey’s HSD post hoc test where Levene’s test *p* ≥ 0.05; Games-Howell post hoc test where Levene’s test *p* < 0.05). Values in same column with different superscript letters (x,y) between cold-pressed and refined type of the same oil differ significantly (Student’s *t*-test, *p* ≤ 0.05).

### 3.7 Effect of thermal treatment on colour of cold pressed and refined oils

Figure 4 represents the colour properties of thermally treated CP and RF oils. Non-heated samples of palm (RF), coconut (CP), and sunflower oil (CP & RF) exhibit higher lightness (L*) values compared to other oils. The L* value consistently decreased with continuous heating across oils, with CP sesame oil showed the most pronounced reduction of L* value.

**Figure 4:**
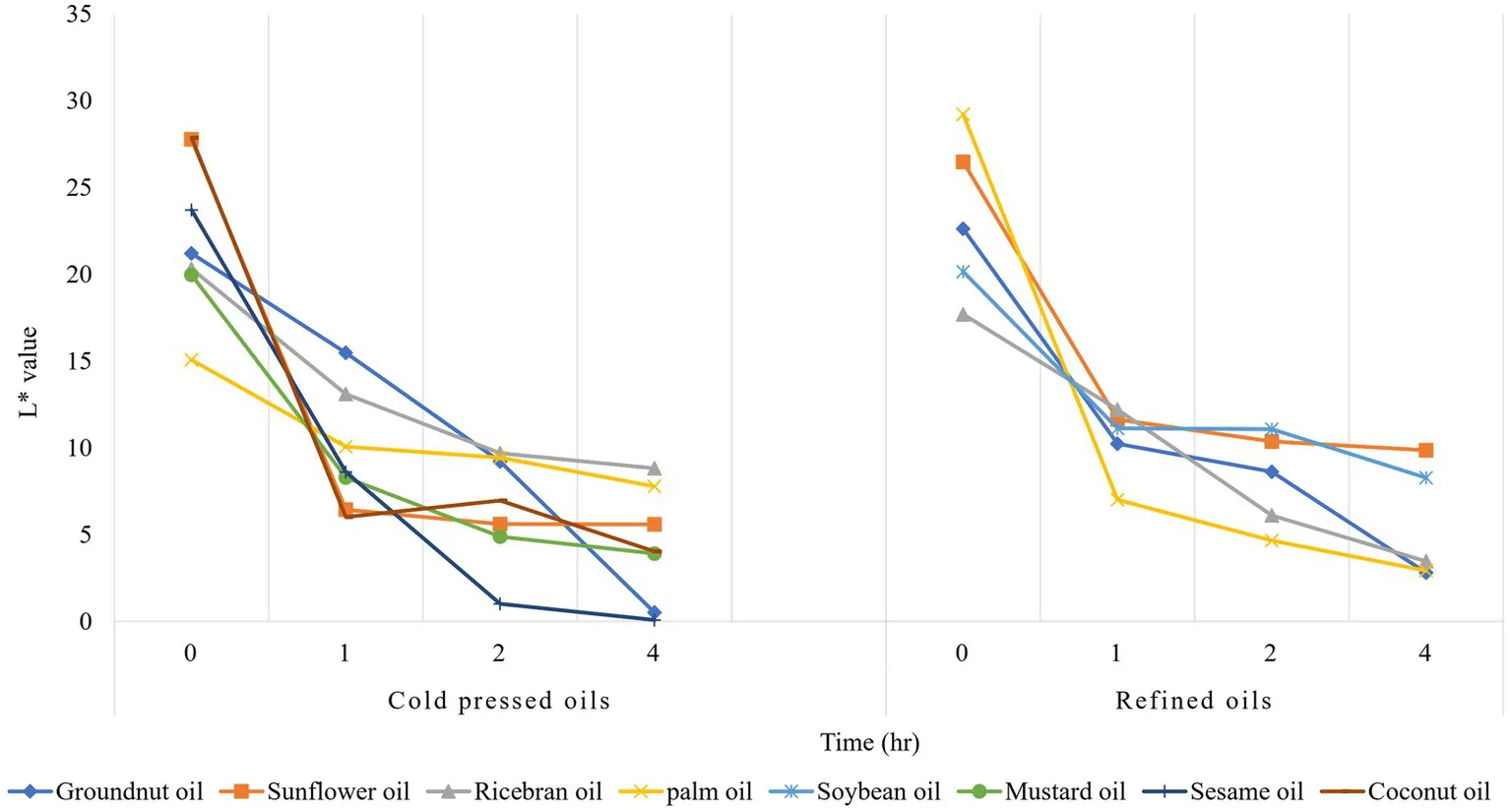
Effect of thermal treatment on colour of cold-pressed and refined oils.

### 3.8 Effect of thermal treatment on refractive index of cold pressed and refined oils

Table 4 shows the refractive index (RI) of all CP and RF thermally treated and non-heated oils. Among non-heated oils, RF rice bran and soybean oils exhibited the highest RI (1.472), while CP coconut oil showed the lowest (1.452). Groundnut (CP & RF) and palm oil (CP & RF) are displayed an initial decline in RI after 1 h of thermal exposure, followed by an increase at later stages. In contrast, most of the oils showed progressive rise in RI with heating duration, except for CP rice bran, mustard and RF soybean oil, which exhibited fluctuating pattern. The CP rice bran oil RI increased in the 1 h, declined at 2 h, and rose again by 4 h of heating. The RI of CP mustard and RF soybean oil values are increased till 2 h of heating and dropped in further thermal exposure. Palm oil showed relatively minor RI modification, (CP, 1.465 ± 0.003–1.467 ± 0.002; and RF, 1.466 ± 0.003–1.466 ± 0.001) after 4 h continuous thermal exposure.

**Table 4:** Effect of thermal treatment on the refractive index of cold-pressed and refined oils.

Oil name	Oil type	0 h	1 h	2 h	4 h
Groundnut oil	Cold pressed	1.466 ± 0.002^a,1,x^	1.465 ± 0.003^a,1-3,x^	1.474 ± 0.005^a,1,x^	1.474 ± 0.004^a,1,x^
	Refined	1.467 ± 0.001^ab,1,x^	1.465 ± 0.003^a,1-3,x^	1.470 ± 0.001^a,2,y^	1.475 ± 0.004^a,1,x^
Sunflower oil	Cold pressed	1.470 ± 0.003^a,1,x^	1.473 ± 0.001^b,2,x^	1.475 ± 0.001^a,1,x^	1.476 ± 0.003^a,1,x^
	Refined	1.470 ± 0.003^a,1,x^	1.473 ± 0.001^a,2,x^	1.473 ± 0.001^a,1,x^	1.476 ± 0.003^a,1,x^
Rice bran oil	Cold pressed	1.470 ± 0.004^a,1,x^	1.474 ± 0.002^a,1,x^	1.472 ± 0.001^a,1,x^	1.474 ± 0.001^a,1,x^
	Refined	1.472 ± 0.009^a,1,x^	1.473 ± 0.001^b,2,x^	1.473 ± 0.001^b,1,x^	1.476 ± 0.001^a,1,x^
Palm oil	Cold pressed	1.465 ± 0.003^a,1,x^	1.462 ± 0.003^a,2-3,x^	1.465 ± 0.002^a,2,x^	1.467 ± 0.002^a,1-2,x^
	Refined	1.466 ± 0.003^a,1,x^	1.462 ± 0.003^a,1-3,x^	1.464 ± 0.003^a,1-2,x^	1.466 ± 0.001^a,2,x^
Mustard oil	Cold pressed	1.468 ± 0.002^a,1^	1.475 ± 0.003^a,1^	1.477 ± 0.004^a,1^	1.475 ± 0.002^a,1^
Sesame oil	Cold pressed	1.468 ± 0.004^a,1^	1.472 ± 0.001^a,3^	1.473 ± 0.001^a,1^	1.473 ± 0.001^a,2^
Soybean oil	Refined	1.472 ± 0.001^a,1^	1.476 ± 0.001^a,1^	1.476 ± 0.003^a,1^	1.475 ± 0.001^a,1^
Coconut oil	Cold pressed	1.452 ± 0.003^a,2^	1.454 ± 0.004^a,2–3^	1.455 ± 0.003^a,2^	1.456 ± 0.004^a,2^

Values are mean±SD (*n* = 3). Values in same row with different superscript letters (a–d) across heating durations differ significantly and values in same column with different superscript numbers (1–12) across oils differ significantly (two-way ANOVA with Tukey’s HSD post hoc test where Levene’s test *p* ≥ 0.05; Games-Howell post hoc test where Levene’s test *p* < 0.05). Values in same column with different superscript letters (x,y) between cold-pressed and refined type of the same oil differ significantly (Student’s *t*-test, *p* ≤ 0.05).

## 4 Discussions

Thermal treatment exhibited profound effect on the physicochemical properties and nutritional quality of edible oils. Continuous exposure to high temperature initiates complex oxidative reactions that alter fatty acid composition and generate secondary oxidation products. These changes not only reduced levels of TPUFA, but also promote the formation of TTFA, CD, and CT, thereby compromising both the stability and health value of oils. Quality indices such as PV, SV, FFA, AV, IV, RI, and colour parameters serve as reliable markers to track changes, reflecting the progressive deterioration of oils under continuous heating. Thermal stress reshapes the chemical profile of oils, highlighting variations in oxidative susceptibility, degradation, and overall stability. However, nine units of same oils were pooled into a single composite sample. The findings of present study, therefore reflect the thermal response of the composite sample constructed for this experiment, not the variability of individual commercial products or brands.

### 4.1 Effect of thermal treatment on fatty acid composition of cold pressed and refined oil

Exposure to high thermal processing markedly influence the fatty acid profile of edible oils. In the present study, heating altered the fatty acid profile across all oils with consistent depletion of TPUFA coupled with relative enrichment of TMUFA (except in coconut oil) and TSFA. This pattern reflects oxidative cleavage of TPUFA and proportional redistribution within the fatty acid pool, result in relative increase in monounsaturated fatty acids (MUFA) and saturated fatty acids (SFA) (34). Edible oil degradation during frying is a complex process that involves various chemical reactions, including hydrolysis, oxidation, isomerization, and polymerization (35). Thermal oxidation and polymerization emerged as the primary causes of TPUFA reduction, producing hydroperoxides and secondary oxidation products, that may responsible for both the reduction and transformation of polyunsaturated fatty acid (PUFA) (36–38). The carbon double bonds in PUFA are the principal sites of autoxidative attack, and preferentially deplete during heating, while SFA remains comparatively unreactive (39). Depletion of oxidative liable unsaturated fatty acids can produce relative enrichment of SFA fraction (40). The MUFA also known to be susceptible to oxidation during heat exposure (41), this trend corroborated with the present study, where coconut oil TMUFA content decreased significantly from 7.71 ± 0.13 to 0.62 ± 0.03%. TPUFA and TMUFA reduction in the coconut oil reflect the low baseline of unsaturated fatty acid content. Mustard oil exhibited higher content of TMUFA than TPUFA, upon heating, TMUFA increased from 62.09 ± 0.20 to 69.82 ± 0.01%, while TPUFA decreased from 30.64 ± 0.15 to 17.31 ± 0.04%, consistent with the proportional increase result from the relative loss of TPUFA rather than net MUFA formation. The PUFA have bis-allylic hydrogens with much weaker carbon, hydrogen bonds than the MUFA, so, heat driven free radical oxidation attack and degrade PUFA far faster, and break them in to hydroperoxides (34, 42). Progressive increase in TTFA was observed in all oils throughout the thermal exposure. The PUFA rich oils such as soybean and sesame oils exhibited with higher accumulation of trans fatty acid (TFA) formation, with 0.76 ± 0.08 to 5.88 ± 0.04% and 0.16 ± 0.01 to 4.05 ± 0.01%, respectively after 4 h of heating. A similar study was done at 220 °C was produced 3.70 ± 0.75 g/100 g of TTFA in soybean oil (21). During thermal exposure, oxidative radicle attack on the carbon double bond (C=C) of unsaturated fatty acid, break the π bond, generally allyl radicle. These unstable radicles undergo free rotation around carbon single bond (C-C). Result in formation *trans* configured doble bond, which is thermodynamically more stable than the *cis* (34). High intake of TFA increase the risk of coronary heart disease, development of dyslipidemia, cardiovascular disease (CVD), and metabolic dysfunction-associated steatotic liver disease, formerly known as non-alcoholic fatty liver disease (13).

### 4.2 Effect of thermal treatment on peroxide value of cold pressed and refined oils

The PV data generated in this study, spanning twelve oils across four time points under continuous thermal exposure at 230 ± 5 °C relevance to the real-world conditions, revealed distinct oil specific oxidative patterns that provide comparative insights beyond those available from single oil or lower temperature studies reported in the existing literature. The PV is a key indicator of oxidative spoilage in edible oils, assess the degree of peroxides and hydroperoxides during the early stages of lipid oxidation (7). Differences in the PV progression among oils reflect variation in production processing (7). Previous studies have reported a decline in a PV during prolonged heating (43, 44), which aligns with the decomposition of lipid peroxides into secondary oxidation products (45, 46). Lipid oxidation begins when thermal energy, oxygen exposure, and light initiate a radical mediated chain reaction within the lipid matrix, resulting in accumulation of lipid hydroperoxides as primary oxidative intermediates (47). These unstable hydroperoxide molecules subsequently fragment through thermal cleavage pathways, releasing free radicals that undergo allylic rearrangement and hydrogen abstraction to yield conjugated diene (CD) and conjugated triene (CT) structures, alongside a spectrum of secondary carbonyl species including saturated and unsaturated aldehyde, ketones, alcohols and reactive hydrocarbons radicals (37, 38). In the present study, Groundnut (CP & RF), soybean (RF), sesame (CP) and coconut (CP) exhibited an initial upward trend then decreased during continuous heating and remaining oils are exhibited downward trend. The decline of PV during prolonged heating reflects the preferential decomposition of hydroperoxides with a concurrent rise in CD and CT, mark the transition from primary to secondary oxidation. This was corroborating with CD and CT values (Table 3) of present study, which are increased with heating exposure increased. Hydroperoxides breakdown generates more stable structures containing trans and conjugated double bonds after heat treatment, it indicates the advanced stage oxidation (48, 49). Moreover, oxidative stability is strongly influenced by frying conditions, oil quality, interaction with food matrix (50). According to the Codex Alimentarius Standard 210, the PV should remain below 10 mEq O_2_/kg (51). Literature has documented that elevated peroxide value is linked to health consequences stemming from oxidative stress such as endothelial dysfunction and mutagenic alterations (52). However, PV alone is insufficient to fully characterize oil total oxidation without concurrent oxidation markers such as p-anisidine value (p-AV), total oxidation value (TOTOX), total polar compounds (TPC), polymerized triacylglycerols, aldehydes, and thiobarbituric acid reactive substance (TBARS) and toxicological data, which represents a limitation of the present study.

### 4.3 Effect of thermal treatment on saponification value of cold pressed and refined oils

The SV represents the mg of KOH required to hydrolyze 1 g of oil, reflecting the average fatty acid chain length. Oils with shorter chains exhibit higher SV, whereas longer chain lipids yield lower values (53). Previous studies have reported various thermal responses of oils. Khaled et al. (31), observed a significant rise in SV during initial heating across various oils, followed by a decline with continuous heating, most notably in palm and soybean oil. In contrast, Alakowe (54) reported a continuous decrease in SV of oils, while (55) noted an initial drop in groundnut oil SV, followed by an increase with further heating. The present findings align partly with these reports, sunflower oil showed the rise then decline pattern, CP coconut oil exhibited a continuous rise with chain scission in to shorter fatty acids. Rice bran oil revealed contrasting trend between the CP and RF, and greater fluctuation was observed throughout heating exposure. This patterns in RF rice bran oil parallels its CD profile (Table 3), which also declines at 1 h before rising at 2 and 4 h, suggesting transit induction period conferred by its native antioxidants (oryzanol and tocopherols) that temporarily delays hydrolytic and oxidative changes before degradation accelerate with continued heating (56). The SV fluctuations during oil heating reflect the competing kinetics of fatty acid liberation and removal. In the initial heating phase, SV increase as thermal energy and residual moisture catalyze the hydrolytic rupture of triglyceride ester bonds, liberate free fatty acids and elevate KOH requirement (36). However, during extended heating, SV may plateau or decline as multiple loss pathways become dominant, with free fatty acids volatilize and escape the oil due to their higher vapor pressure compared to intact triglycerides (39). These variations highlight that SV responses are oil specific, reflecting differences in fatty acid composition.

### 4.4 Effect of thermal treatment on free fatty acid and acid value of cold pressed and refined oils

The AV quantifies the mg of KOH required to neutralize FFA in 1 g of oil, while FFA denotes the weight percentage of a specific fatty acid, typically expressed as oleic acid. AV is widely recognized as a marker of oil degradation resulting from hydrolytic activity or enzymatic action (57), whereas elevated FFA is closely associated with the generation of undesirable flavour and odour that diminish sensory quality and consumer acceptability (58). Among all oils, substantial reduction in FFA and AV was observed in CP palm oil declining from 8.26 ± 0.07 to 0.18 ± 0.01 (% oleic acid) and 16.44 ± 0.13 to 0.37 ± 0.01 mg KOH/g, respectively. The marked decrease in FFA and AV in palm oil (CP) after 4 h reflects the volatilization of FFA, which possess higher vapor pressure than triglycerides and escape from the oil matrix at elevated temperature (39). This mechanism parallels industrial deodorization, where palm oil refined at 230–260 °C achieve final FFA content below 1% (58). Such decline is attributed to esterification with glycerol and volatilization of short chain fatty acids under elevated thermal conditions (59). Prior studies corroborate these findings, showing that heating palm, groundnut and soybean oils at thermal treatment above 200 °C showed substantial FFA reduction via thermoxidisation and volatilization (7, 21). Sunflower oil heated at 99 °C showed decreased AV during prolonged exposure (60). In present study, consistent with these reports, demonstrated progressive decrease in FFA and AV across all oils, particularly rice bran oil (CP & RF) showing sharp decline to negligible levels after 1 to 2 h, and coconut and sesame oil reaching minimal value after 4 h of heating. Continuous high thermal treatment for 4 h provide sufficient time for cumulative FFA volatilization to outpace FFA generation, which result in net AV decrease (61).

### 4.5 Effect of thermal treatment on iodine value of cold pressed and refined oil

The IV results were expressed as grams of iodine absorbed by 100 g of oil, indicating the degree of unsaturation in edible oil (62). Previous studies have reported that heating reduces linoleic acid (C18:2) in oils, thereby lowering IV through enhanced thermo-oxidative reactions (63). This decline is primarily attributed to the breakdown of double bonds through oxidative degradation, molecular scission, and polymerization processes (36, 64). Consistent with this mechanism, the present study demonstrated progressive reduction in IV across all oils during heating. Similar trend was observed in the both type of groundnut oil CP with 8.82 and RF with 12.10% of reduction. RF soybean and rice bran oil retained relatively stable values after 4 h of heat treatment. The CP coconut oil was showed maximum IV decline, which was contradicting with the residual unsaturated fatty acid content of chromatographic results (Table 1). For CP coconut oil after 4 h heat treatment, IV was calculated manually following the AOCS (1998) formula, which showed the result as 0.86 g I_2_/100 g. The steric bulk of iodine restricts its ability to assess and saturate conjugated double bond systems, particularly when multiple conjugations are present in a single fatty acid molecule. As a consequence, IV obtained through the titration method may underestimate the lower unsaturation value compared to values derived from gas chromatography, which directly quantifies individual fatty acid methyl ester profile (65).

### 4.6 Effect of thermal treatment on conjugated dienes and trienes of cold pressed and refined oils

The CD and CT are closely associated with oxygen uptake and peroxide generation in oils, serving as a reliable marker of advanced lipid oxidation. During thermal treatment, linoleic acid undergoes structural rearrangements within its pentadienoic moiety, which promotes the formation of CD and CT (66). In the present study, CD values showed a clear, progressive increase during the initial stages of thermal treatment, consistent with earlier reports in sunflower oil (67). Rice bran oil (CP & RF) exhibited an initial decline in CD after 1 h, followed by a renewed increase with continuous heating, while palm oil (CP & RF) showed lowest CD after 1 h, likely due to its high saturated fatty acid content. The oils rich in PUFA produce higher levels of CD and CT during heating due to oxidative susceptibility. The CT levels rise sharply onset of heating, then decline as they convert into polymeric compounds during continuous heating (68).

### 4.7 Effect of thermal treatment on colour of cold pressed and refined oils

Edible oil colour is chiefly determined by natural pigments such as carotenoids, chlorophylls, as well as processing-derived pigments formed through oxidative polymerization and non-enzymatic browning (69). During thermal treatment, the unsaturated fatty acids in triacylglycerols undergo oxidative polymerization, generate polar compounds including monomers, dimers, and oligomers that contribute to oil darkening (70). In the present study L* value consistently decreased with continuous heating across oils, with sesame oil showing the greatest reduction. Similar trend was reported in soybean, palm, rice bran, and mustard oils during thermal exposure (71). Thermal oxidation and auto-oxidation of phospholipids further accelerate pigment loss and darkening. Oil exposure to the high thermal treatment darkens the colour due to degradation of natural colour, maillard reaction between the amino compounds and the lipid oxidation carbonyl produce the brown melanoidin polymers, and condensation of the secondary oxidation products (aldehydes and ketones) into higher molecular weight brown polymers through aldol condensation and radical mediated polymerisation (37–39). Oils like coconut and safflower are particularly susceptible to these changes, where heating significantly reduces pigment content and thereby diminishes oil L* value (69).

### 4.8 Effect of thermal treatment on refractive index of cold pressed and refined oils

The RI is a sensitive parameter that reflects compositional changes in edible oils during processing and storage. In the present study, fresh RF rice bran and soybean oil existed the highest RI values (1.472), while CP coconut oil showed the lowest (1.452). These findings are consistent with the earlier reports of RI in rice bran oil (72), and coconut oil (1.454) (73). The RI ranges of groundnut (1.460–1.465), sunflower (1.461–1.468), rice bran (1.460–1.473), palm (1.454–456), mustard (1.461–1.469), sesame (1.465–1.469), soybean (1.466–1.470) and coconut oil (1.448–1.450) recommended by the Food and Agriculture Organization (FAO)/World Health Organization (WHO) (74). Initial RI of groundnut oil (CP, 1.466; RF, 1.467), sunflower oil (CP, 1.470; RF, 1.470), palm oil (CP, 1.465; RF, 1.466) RF soybean oil (1.472) and CP coconut oil (1.452) showed higher values, and remaining oils fell within the recommended range of FAO/WHO, confirming their initial quality. An increase in RI generally indicates alterations in unsaturation and the formation of advanced oxidation products such as CD and CT from thermally induced rearrangement of double bonds. This conjugation enhances the electronic density and polarizability of the oils, elevate its RI. During advanced heating, polymerization of oxidized lipids forms high molecular-weight dimers and oligomers with a greater electron density than monomeric triglycerides, further increase the RI (73, 75). The magnitude of RI change correlates directly with oxidation progression and conjugation formation, making refractive index a practical, non-destructive quality indicator for thermal degradation of edible oils (77). Measuring RI can help in quality control and spotting adulteration in edible oils (76).

### 4.9 Principal component analysis

Principal component analysis (PCA) was performed to evaluate the differentiation and chemical relationships among the edible oil samples across various thermal exposure. Oil categories with codes as followed, Groundnut CP (GrC), Groundnut RF (GrR), sunflower CP (SuC), sunflower RF (SuR), rice bran CP (RiC), rice bran RF (RiR), palm CP (PoC), palm RF (PoR), mustard CP (MuC), sesame CP (SeC), soybean RF (SoR) and coconut CP (CoC). Number (0, 1, 2 and 4) beside the oil code represent the hours of heating exposure. The first two principal components (PC1 and PC2) explained a cumulative variance of 64.4% (PC1: 38.7 and PC2: 25.7%). As illustrated in Figure 5, PC1 was primarily driven by the fatty acid saturation profile and optical properties, showing strong positive loadings for IV (0.43), RI (0.42), and TPUFA (0.36), and strong negative loadings for TSFA (−0.44). This effectively separated highly saturated oils from polyunsaturated oils along the horizontal axis. In contrast, PC2 was driven by hydrolytic and isomerization parameters, with strong positive loadings TTFA (0.49), SV (0.35) and CT (0.31) and negative loading for TMUFA (−0.46) and AV (−0.38). The dispersion of sample points along PC2 highlights the progressive chemical changes and stability differences occurring across the thermal exposure intervals. Overall, it was observed that the non-heated (0 h) oils displayed greater proximity with L*, TPUFA, TMUFA, IV, RI, and AV, while 2 and 4 h thermally treated oils associated with the TTFA, CD and CT. Coconut and palm oils were clustered distinctly from the remaining oils, irrespective of heating duration. Their separation was strongly associated with TSFA.

**Figure 5:**
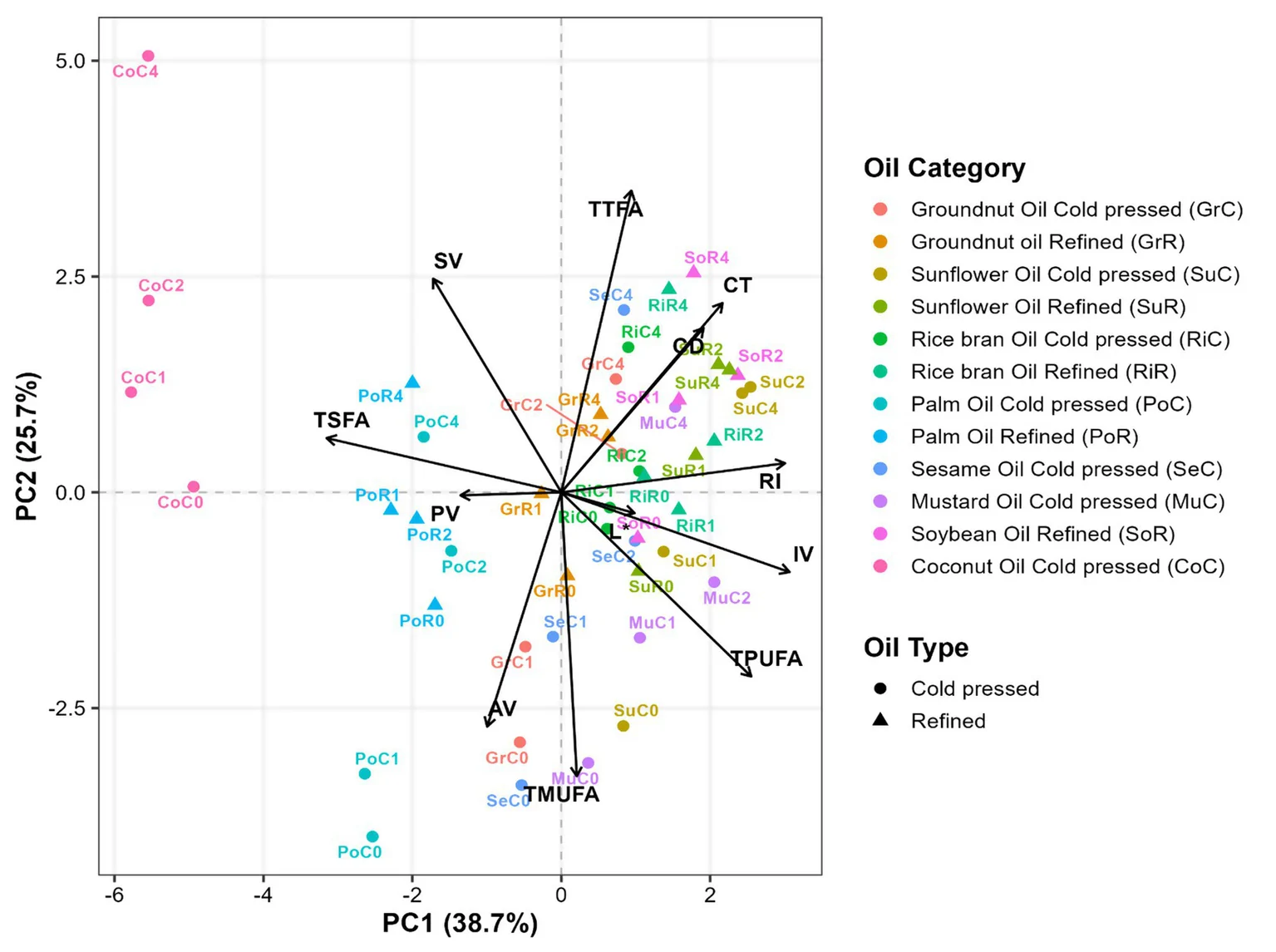
Distribution of Principle components of thermal treated cold pressed and refined oils.

## 5 Conclusion

Continuous high thermal processing degrades oil through two stage pathways: early hydrolysis and primary oxidation which raise FFA, AV and PV, followed by secondary oxidation and isomerization at longer exposure, raise CD, CT, TFA, alongside progressive darkening of oil. Based on the fatty acid, PV and SV trend measured in this study, saturated oils (coconut and palm) showed comparatively slower thermo-oxidative change under extended heating, while PUFA rich oils (soybean and sesame oil) showed faster TFA accumulation under continuous heating, indicating greater susceptibility to thermal isomerization than the more saturated oil tested. Among all oils tested, rice bran oil both types (CP & RF), exhibited comparatively stable physicochemical behavior across the measured parameters under continuous heating. These findings indicate that fatty acid unsaturation, rather than processing method, is the primary determinant of thermo-oxidative stability. This study does not comprise the same botanical oil types (CP and RF) of all oils, so present design cannot isolate a general effect of processing type. The CP and RF comparison was interpreted for matched botanical pairs (groundnut, sunflower, rice bran and palm oil), while mustard, sesame, coconut, and soybean were regarded as characterization of individual oils. However, definitive frying safety recommendations require additional oxidation and toxicological endpoints (e.g., total polar compounds, TOTOX, polymerized triacylglycerols) were not measured. These findings may nonetheless inform future, more comprehensive research towards oil specific frying guidance relevant to street food and industrial cooking practices.

## Data Availability

The original contributions presented in the study are included in the article/supplementary material, further inquiries can be directed to the corresponding author.

## Glossary

ANOVA: Analysis of variance
AV: Acid value
BHT: Butylated hydroxy toluene
CD: Conjugated diene
CLR: Centered log-ratio
CoC: Coconut cold-pressed
CP: Cold-pressed
CVD: Cardiovascular disease
CT: Conjugated triene
EFAs: Essential fatty acids
FAO: Food and agriculture organization
FFA: Free fatty acid
FID: Flame ionization detector
GC: Gas chromatography
GrC: Groundnut cold-pressed
GrR: Groundnut refined
HCl: Hydrochloric acid
IV: Iodine value
KI: Potassium iodide
KOH: Potassium hydroxide
MUFA: Monounsaturated fatty acids
MuC: Mustard cold-pressed
p-AV: p-anisidine value
PCA: Principal component analysis
PoC: Palm cold-pressed
PoR: Palm refined
PUFA: Polyunsaturated fatty acids
PV: Peroxide value
RF: Refined
RiC: Rice bran cold-pressed
RiR: Rice bran refined
RI: Refractive index
SD: Standard deviation
SeC: Sesame cold-pressed
SFA: Saturated fatty acids
SoR: Soybean refined
SuC: Sunflower cold-pressed
SuR: Sunflower refined
SV: Saponification value
TFA: Trans fatty acid
TMUFA: Total monounsaturated fatty acids
TOTOX: Total oxidation value
TPC: Total polar compounds
TPUFA: Total polyunsaturated fatty acids
TSFA: Total saturated fatty acids
TTFA: Total trans fatty acids
TBARS: Thiobarbituric acid reactive substances
WHO: World Health Organization
